# Leveraging Vaccines to Reduce Antibiotic Use and Prevent Antimicrobial Resistance: A World Health Organization Action Framework

**DOI:** 10.1093/cid/ciab062

**Published:** 2021-01-25

**Authors:** Johan Vekemans, Mateusz Hasso-Agopsowicz, Gagandeep Kang, William P Hausdorff, Anthony Fiore, Elizabeth Tayler, Elizabeth J Klemm, Ramanan Laxminarayan, Padmini Srikantiah, Martin Friede, Marc Lipsitch

**Affiliations:** 1 Immunization, Vaccines and Biologicals (IVB), World Health Organization (WHO), Geneva, Switzerland; 2 Division of Gastrointestinal Sciences, Christian Medical College, Vellore TN, India; 3 PATH, Washington, District of Columbia, USA Faculty of Medicine, Université Libre de Bruxelles, Brussels, Belgium; 4 Centers for Disease Control and Prevention, Atlanta, Georgia, USA; 5 Antimicrobial Resistance Division, World Health Organization (WHO), Geneva, Switzerland; 6 Wellcome Trust, Bloomsbury, London, United Kingdom; 7 Center for Disease Dynamics, Economics & Policy, Washington District of Columbia, USA; 8 Bill and Melinda Gates Foundation, Seattle, Washington, USA; 9 Harvard T. H. Chang School of Public Medicine, Boston, Massachusetts, USA

**Keywords:** vaccines, AMR, WHO

## Abstract

This Action Framework identifies priority actions to prevent antimicrobial-resistant (AMR) through expanding the use of licensed vaccines, developing new vaccines that contribute to the prevention and control of AMR, and expanding knowledge about the impact of vaccines on AMR.

Antimicrobial resistance (AMR) threatens to undermine the effectiveness of antimicrobials and undo progress made against infectious diseases. Pathogens resistant to all classes of antimicrobials can be found throughout the world, and the incidence of infections with resistant pathogens is growing [1]. The risk of AMR infection is increased in clinical care settings, where the use of antibiotics is frequent and pathogen transmission high, threatening the continuity of safe provision of routine care and surgical procedures [2]. The second- or third-line drugs used to treat resistant pathogens are typically more expensive or require administration in hospital settings, and sometimes are less effective or have serious side effects (such as colistin), making them less accessible to people living in low- and middle-income countries (LMICs) [3]. Unless there is a rapid and multifaceted response to prevent and control AMR, very significant economic costs from lost productivity and social disruption by 2050 are highly likely [4]. The Global Action Plan on Antimicrobial Resistance [5] and other reports [6] state that addressing AMR will require improvements in infection prevention, antimicrobial stewardship, and antimicrobial discovery (Figure 1).

**Figure. 1.**
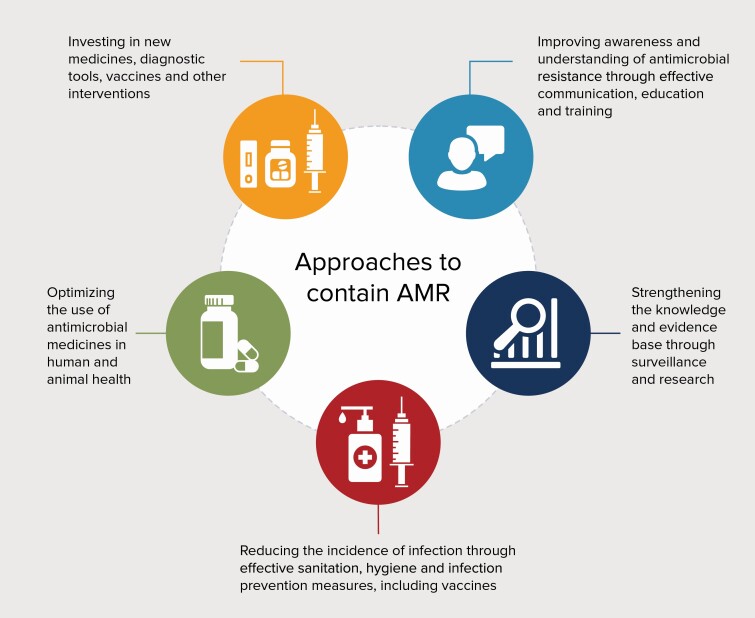
Strategic objectives of the Global Action Plan on Antimicrobial Resistance

The discovery and use of new antibiotics constitute a complex economic and scientific challenge. Few antibiotic classes have been developed in the past half-century [7], and resistant isolates can emerge in the relatively short term, jeopardizing effective and sustainable use [8]. Antimicrobial stewardship encourages more responsible use of antimicrobials and minimizes the selection pressures that drive the development of resistance. Infection prevention reduces the need for antibiotic treatment. Vaccines can play a key role in reducing the need for antibiotic treatment and the selection pressure that drive the development of resistance.

## Vaccines Contribute to the Battle Against AMR by Preventing Infections and by Reducing Antimicrobial Use

The most direct way in which vaccines contribute to prevention and control of AMR is by reducing the incidence of disease from resistant pathogens [9]. A study in South Africa demonstrated a 67% reduction in penicillin resistant invasive pneumococcal disease episodes in the group that received pneumococcal conjugate vaccine 9 (PCV9) compared with controls [10]. A similar impact of PCV has been observed in other trials [11]. The introduction of typhoid conjugate vaccine (TCV) is expected to avert 44% of typhoid cases, of which 35% are resistant to antibiotics [12]. The importance of protecting against resistant strains of *Salmonella typhi* led to an introduction of TCV in children to help control the spread of extensively drug-resistant typhoid [13].

By reducing the incidence of diseases, vaccines also reduce antibiotic use. Because the clinical presentations of many infections do not appreciably differ whether caused by bacteria or viruses, and antibiotic use is often presumptive, vaccines that reduce the incidence of syndromic diseases may also reduce antibiotic use. With current vaccine coverage, pneumococcal and rotavirus vaccines prevent 23.8 million and 13.6 million episodes of antibiotic-treated illness, respectively, among children in LMICs each year [14]. Influenza vaccines reduces days of antibiotic use in adults by 28.1% [15].

Vaccines that reduce the incidence of antibiotic use can contribute to reducing selection for AMR in the target pathogen (for bacterial vaccines) as well as in bystander bacterial species, often present in the normal flora, which can transmit and cause disease (*Escherichia coli*, *Klebsiella pneumoniae*, or *Staphylococcus aureus* [16]).

Some vaccines could potentially reduce antibiotic use to an extent that exceeds the causal fraction of the disease syndrome resulting from the vaccine target pathogen. A vaccine effective against group A *Streptococcus* would reduce the need for presumptive antibiotic treatment for pharyngitis. Vaccines effective against key pathogens causing a given clinical syndrome might ultimately result in synergistic effects on antimicrobial use and therefore less resistance.

The World Health Organization (WHO) created a list of antibiotic-resistant bacteria for which new antibiotics are urgently needed [17] and the Wellcome Trust assessed the feasibility of developing vaccines for these pathogens [18]. Pathogen clusters were identified for which different interventions are required: increase uptake, bring to market, advance early research and development (R&D), collect data, and explore alternatives. Similarly, the World Organization for Animal Health prioritized vaccine use for animal health [19, 20]. Such systematic approaches to collect evidence, prioritize, and understand the full potential impact of interventions, including vaccines to contain AMR, are essential to inform the value proposition, justify the need for investment, define the product use case, and inform decision making. Economic, social, and equity effects of vaccines and alternatives on AMR must be assessed to understand their value and be promptly and transparently disseminated. Although better evidence will enhance confidence in decisions, the urgency of the AMR threat, combined with the long time lag for some types of investments to pay off, demands that we make decisions and investment based on currently available data.

## PURPOSE AND METHODOLOGY

This Action Framework intends to guide vaccine stakeholders to maximize the impact of vaccines in preventing AMR. It is a result of a collaboration between WHO, the Wellcome Trust, the Bill & Melinda Gates Foundation, and the Center for Disease Dynamics, Economics & Policy and was developed through a consensus-building consultative process involving experts from academic institutions, country representatives, nongovernmental organizations, and the pharmaceutical industry. A formally constituted Vaccines for AMR WHO working group of experts on AMR and immunization provided suggestions throughout the process, and the Framework was drafted following a consultation in February 2019. The document has been circulated for comment and public review. The objective of this document is to support an alignment of activities among vaccine and AMR partners, and structure and articulate key priority actions with a goal to expedite the understanding, development, and use of vaccines against AMR. The document complements the global immunization strategy, the Immunization Agenda 2030: A Global Strategy to Leave No One Behind [21].

## The Action Framework

This Action Framework supports a strategic vision for vaccines to contribute fully, sustainably, and equitably to the prevention and control of antimicrobial resistance by preventing infections and reducing antimicrobial use. To achieve this vision, we propose 3 goals with appropriate objectives (Panel 1) and priority actions below.

**Panel 1. Goals and Objectives to Maximize the Impact of Vaccines Against Antimicrobial Resistance**

**Goal 1. Expand the use of licensed vaccines to maximize impact on AMR**

Objective 1. Increase coverage of vaccines with impact on AMR

Objective 2. Update recommendations and normative guidance in both the vaccine and AMR sectors to include the role of vaccines to control AMR

Objective 3. Improve awareness and understanding of the role of vaccines in limiting AMR through effective communication, education, and training

**Goal 2. Develop new vaccines that contribute to prevention and control of AMR**

Objective 4. Bridge the funding gap for R&D of new vaccines with potential for global AMR impact

Objective 5. Develop regulatory and policy mechanisms to accelerate approval and use of new vaccines that can reduce AMR

**Goal 3. Expand and share knowledge of vaccine impact on AMR**

Objective 6. Improve methodologies and increase collection and analysis of data to assess vaccine impact on AMR, including antimicrobial use

Objective 7. Develop estimates of vaccine value to avert the full public health and socioeconomic burden of AMR

Abbreviations: AMR, antimicrobial resistance; R&D, research and development.

### Goal 1. Expand Use of Licensed Vaccines to Maximize Impact on AMR

Expanding the use of licensed vaccines will require reaching current uptake targets and setting and achieving ambitious coverage targets for newly licensed vaccines with the potential impact on AMR.

#### Objective 1. Increase Coverage of Vaccines With Impact on AMR

Maximizing the impact of immunization on AMR will depend on the successful implementation of a global strategy with an integrated Action Framework linking immunization to primary health care and universal health coverage.

##### Priority Actions.

1a: Countries should implement existing vaccine-related recommendations of the Global Action Plan on AMR. Priority should be given to completion of the full basic series of PCV, *Haemophilus influenzae* type B vaccine, rotavirus vaccine, and measles-containing vaccines as well as increasing coverage for influenza and TCV.

1b: Donors, countries, and other health payers should maintain and expand immunization financing and strengthen capacities, ensuring affordable supply, functional delivery systems, and programmatic sustainability. These should also support vaccine safety and effectiveness monitoring.

#### Objective 2. Update Recommendations and Normative Guidance in Both the Vaccine and AMR Sectors to Include the Role of Vaccines to Control AMR

New activities are needed to expand the impact of vaccines on AMR. Expanding the benefits of immunization throughout the life course will play a major role. When research and epidemiologic data emerge that justify changes in optimal vaccine use, revised recommendations should be developed, including situations in which vaccines are used to protect the effectiveness of antimicrobials. Specific vaccine use recommendations could also be developed for vulnerable groups who, for medical reasons, use antibiotics chronically or frequently, or who are at increased risk of exposure to drug-resistant microbes, such as healthcare workers.

##### Priority Actions.

2a: Where justified, normative guidance, regulatory indications, policy recommendations, and health regulations for vaccine use should be adapted to account specifically for the use of vaccines to impact AMR.

2b: AMR national action plans and international organizations dedicated to AMR control should consistently include vaccines in the portfolio of interventions planned for use against AMR.

2c: Immunization programs should be strengthened to reach children beyond the first year of life and immunization services broadened to support vaccination with impact on AMR throughout the life course.

2d: As part of a “One Health” perspective, bodies such as WHO, Food and Agriculture Organization, and World Organization for Animal Health, in collaboration with the agricultural industry and animal health stakeholders, should update recommendations and regulations and develop an action plan to maximize the use of animal vaccines to reduce antibiotic use in animals.

#### Objective 3. Improve Awareness and Understanding of the Role of Vaccines in Limiting AMR Through Effective Communication, Education, and Training

The full value of vaccines at the individual and population levels is not completely understood, contributing to low and decreasing confidence and coverage in vaccines in some areas. Communicating the additional benefit of the use of vaccines to fight AMR requires the development of carefully constructed and evaluated communication strategies and tools.

##### Priority Actions.

3a: Countries, funders, and other stakeholders should include the role of vaccines in limiting AMR in their communication materials.

3b: Institutions involved in the vaccine and AMR sectors should develop communication, education, and training materials about the role of vaccines in controlling AMR, targeting audiences ranging from the general public to infectious disease experts.

### Goal 2. Develop New Vaccines That Contribute to Prevention and Control of AMR

New vaccine R&D is an integral part of the Global Action Plan on AMR [5]. Few new antimicrobials have been developed recently or are anticipated to be available soon, and all are threatened by the emergence of resistance [22]. In contrast, vaccines have traditionally had a sustainable impact, with little or no evidence of escape from immunity.

The development and use of new or improved vaccines is of particular importance to prevent diseases becoming difficult to treat or untreatable owing to antimicrobial resistance. Pathogen areas to be prioritized for investments into vaccine R&D should be informed by public value and feasibility assessments, taking into account alternative options such as phage-based medicine or microbiome interventions [23].

#### Objective 4. Bridge the Funding Gap for R&D of New Vaccines With Potential for Global AMR Impact

Investment in the development of new vaccines to affect global health is often impeded by scientific hurdles leading to market failures and decades-long development, high clinical development costs, prolonged licensure, or implementation timelines, as well as lack of recommendations regarding use. These challenges create a perception that vaccines are unattractive business investments. Innovative financing mechanisms channelling substantial public sector funding and private sector investment, push-and-pull incentives, or subscription models may be needed to support new vaccine development, and to bring candidates from discovery through preclinical and clinical testing to licensure, adoption, and implementation in LMICs. Related activities should be monitored and evaluated, in line with the Global Action Plan monitoring and evaluation framework for new products and funding instruments.

##### Priority Actions.

4a: Funders, industry, governments, nongovernmental and supranational organizations, academic institutions, and researchers should consider increasing investments in vaccine candidates with anticipated benefits for AMR.

4b: Funders, including governments and nongovernmental organizations, product development sponsors and industry, should increase financing for late-stage vaccine evaluation, introduction, evaluation of new vaccine effectiveness and impact, and to ensure sufficient manufacturing capacity to meet global needs for vaccines expected to reduce AMR.

#### Objective 5. Develop Regulatory and Policy Mechanisms to Accelerate Approval and Use of New Vaccines That Can Reduce AMR

WHO recommendations inform decision making at multiple levels, including international financing bodies supporting vaccine procurement and distribution. Regulators and policymakers engage in discussions with funders and vaccine developers to prioritize disease areas, product development, investments, and activities, and create scientific consensus. Throughout, specific modalities should be adopted to consider and facilitate vaccine impact on AMR, all along regulatory and policy-making pathways. Vaccines and other AMR-relevant prophylactic interventions (eg, monoclonal antibody, microbiome sparing/restorative preparations) should be considered for inclusion in legislation that incentivize the development of AMR products [24].

##### Priority Actions.

5a: Vaccine development sponsors and regulatory authorities should systematically assess the potential to prevent and control AMR and related data packages generated in clinical development to expand knowledge of investigational product risk–benefit balance.

5b: Vaccine development sponsors and regulators should discuss clinical research requirements for regulatory labelling to include specifications about impact on AMR and antimicrobial use.

5c: Regulators and policymakers should develop means to accelerate access to vaccines of urgent medical need, including impacts on AMR, without jeopardizing the required confidence in safety and efficacy.

5d: WHO, through its Product Development for Vaccines Advisory Committee and Strategic Advisory Group of Experts on Immunization, and other stakeholders who shape progress in vaccine R&D should include evaluation of AMR impact in their product landscape analyses and guidance.

5e: Sponsors of postlicensure vaccine evaluations, such as health–economic impact studies, should discuss with regulators and policymakers, during the approval process, when and how to include evaluation of a vaccine’s potential to reduce antimicrobial use and AMR in these studies.

### Goal 3. Expand and Share Knowledge of Vaccine Impact on AMR

Continuing research is needed to strengthen the knowledge base on the potential role of vaccines in prevention and control of AMR, and this knowledge disseminated to stakeholders. Better estimates of impact will improve policy making and rational prioritization of investments.

#### Objective 6. Improve Methodologies and Increase Collection and Analysis of Data to Assess Vaccine Impact on AMR, Including Antimicrobial Use

There is an urgent need to increase data collection and analysis on the impact of vaccines on AMR [15]. This is particularly relevant to LMICs where issues of both access to and excessive use of antibiotics are important public health concerns.

##### Priority Actions.

6a: Normative bodies should provide guidance for health technology assessment and evaluation of vaccine impact on AMR and antimicrobial use.

6b: Funders and researchers should analyze existing datasets from epidemiologic studies, trials, and routine surveillance to estimate vaccine impact on AMR.

6c: Where relevant, vaccine trials and studies should assess impact on AMR and/or antimicrobial use.

6d: Public health authorities at the global, national, and subnational levels should enhance surveillance data systems to link vaccination data with antimicrobial use and resistance data with the greatest practicable level of geographic and demographic granularity. In resource-limited settings, building capacity for data collection and analysis should be included in immunization and AMR country action plans.

6e: Researchers should continue to generate new evidence on:

• how to use vaccines with the specific aim of controlling drug-resistant pathogens;
• how vaccines can complement other infection control strategies and stewardship efforts to prolong or restore effective use of antibiotics against specific pathogens; and
• socioeconomic and ethical aspects of vaccine impact on AMR.

6f: Researchers and their sponsors should ensure that new data and evidence are made rapidly and publicly available through prompt public posting and scientific publications, preprints, and data-sharing platforms.

#### Objective 7. Develop Estimates of Vaccine Value to Avert the Full Public Health and Socioeconomic Burden of AMR

Decisions about vaccine development, introduction, and use should be informed by estimates of the full value of vaccines, including not just disease impact but also AMR economic burden, antibiotic use, and impact on social justice and equity. Mathematical modelling, multicriteria decision analysis and other methodologies including empirical approaches can be used to inform investment decision making. Through an iterative process, modelling estimates should be regularly refined as empirical data emerge.

##### Priority Actions.

7a: Research funders should support researchers to develop and improve methodologies to estimate impact of vaccines on AMR. Factors such as individual protection, herd immunity, transmission patterns, pathogen carriage rates, bacterial population dynamics, serotype replacement, vaccine-driven reductions in antibiotic use, and the various molecular drivers of resistance should be considered.

7b: Health delivery payers and investors in R&D should develop and use standardized health technology assessments and value–attribution frameworks to inform the estimation of the full value of vaccines to prevent and control AMR.

## Conclusions

Vaccines are already contributing to the battle against AMR through prevention of infections and an associated decrease in antibiotic use. The priority activities outlined in this document provide the opportunity for vaccines to contribute fully, sustainably, and equitably to the prevention and control of AMR as a complementary approach to other AMR reduction efforts.

Increased investments from the private, philanthropic, and public sectors are needed for existing vaccines to increase coverage and to develop new vaccines.

Guidance provided to the AMR and immunization communities should be updated and strengthened to reflect the vision expressed here. Regulatory and policy frameworks should be adapted to support efficient decision making and to maximize vaccine-related opportunities and impact.

Among available vaccines, increased uptake of *Haemophilus influenzae* type B, PCV, TCV, and influenza should be prioritized for impact on antibiotic use and AMR. Among disease areas for which proof-of-concept evidence suggests that vaccine development or improvement is technically feasible, tuberculosis constitutes a major public health emergency and priority for investment. Vaccines against gonococcal infections and enteric diseases resulting from *Shigella*, *E coli*, and nontyphoidal *Salmonella* also constitute priority R&D opportunities.

Development should be accelerated of next-generation vaccines providing expanded strain coverage and durable protection against influenza and pneumococcus, as well as new vaccines against malaria, human immunodeficient virus, respiratory syncytial virus, and group A *Streptococcus*. It may be possible to develop vaccines against other important AMR pathogens such as *S aureus*, *Pseudomonas aeruginosa*, *E coli*, *Campylobacter*, *Helicobacter pylori*, *K pneumoniae*, *Acinetobacter baumannii, Enterococcus faecium*, *Clostridium difficile*, *Chlamydia*, and *Candida*, but in each case technical feasibility remains to be demonstrated.

Across disease areas, key activities to maximize impact on AMR include: further development of innovative technologies, accelerated testing pathways, effectiveness evaluation through pilot implementation, new opportunities for immunization along the life course, access to high-risk groups, and market shaping.

More and better collection and analysis of data on the role of vaccines against AMR across a variety of microbiological, health, and economic sectors are critical. Modelling provides important tools to estimate the full value of vaccines against AMR.

Health interventions and policies depend on public confidence. Advocacy and targeted communication can contribute to increased knowledge and catalyze the action needed to better protect everyone against infections and curb the threat that AMR poses to individuals, societies, and global health.

## Notes

***Author contributions.*** J. V. and M. H. A. organized the expert consultation to develop the manuscript. J. V. and M. H. A. took the lead to write the manuscript. G. K., W. P. H., A. F., E. T., E. J. K., R. L., M. F. , and M. L. contributed to writing the manuscript. All authors provided critical feedback and helped to shape the final version of the manuscript.

***Acknowledgements.*** We thank remaining members of the Vaccines for AMR working group for providing their expertise: Mark Jit (London School of Hygiene and Tropical Medicine [LSHTM], London, UK); Angela Brueggemann (University of Oxford, Oxford, UK), Buddha Basnyat (Oxford University Clinical Research Unit, Kathmandu, Nepal), Gordon Dougan (University of Cambridge, Cambridge, UK), Francis Ndowa (Skin and GU Medicine Clinic, Harare, Zimbabwe), Iruka Okeke (University of Ibadan, Ibadan, Nigeria), David Salisbury (Chatham House, London, UK), Anthony Scott (LSHTM, London, UK), JP Sevilla (Harvard T.H. Chan School of Public Health, Boston, MA, USA), and Lone Simonsen (Roskilde University, Roskilde, Denmark). We extend additional thanks to the following key contributors: Isabel Frost (Center for Disease Dynamics, Economics & Policy, New Delhi, India), Luka Srot (International Federation of Pharmaceutical Manufacturers and Associations, Geneva, Switzerland), Stefano Messori (World Organisation for Animal Health, Paris, France), Wilson Mok (Gavi, Geneva, Switzerland), Holly Prudden (International AIDS Society, Geneva, Switzerland), Robert Taylor (scientific writer, Boston, MA, USA), the WHO IVB Secretariat, Geneva, Switzerland, and the WHO AMR Secretariat, Geneva, Switzerland.

***Financial support.*** This work was funded by the Bill & Melinda Gates Foundation grant OPP1190808.

***Potential conflicts of interests.*** M. L. reports grants from Wellcome Trust, during the conduct of the study; grants from Pfizer, personal fees from Merck, personal fees from Bristol-Meyers Squibb, personal fees from Sanofi Pasteur, grants from National Institutes of Health (US), grants from National Institute for Health Research (UK), grants from Centers for Disease Control and Prevention (US), and grants from Open Philanthropy Project, outside the submitted work. W. P. H. is a patent holder on the 13-valent PCV licensed to Pfizer but receives no royalties as per industry practice (was an employee of Wyeth at the time of invention). All other authors report no conflict of interest.
